# Supplementary figures and images for: Ecological distribution of protosteloid amoebae in New Zealand
Source: PeerJ. 2014 Mar 11;2:e296. doi: 10.7717/peerj.296 (PMC3961141; doi:10.7717/peerj.296)

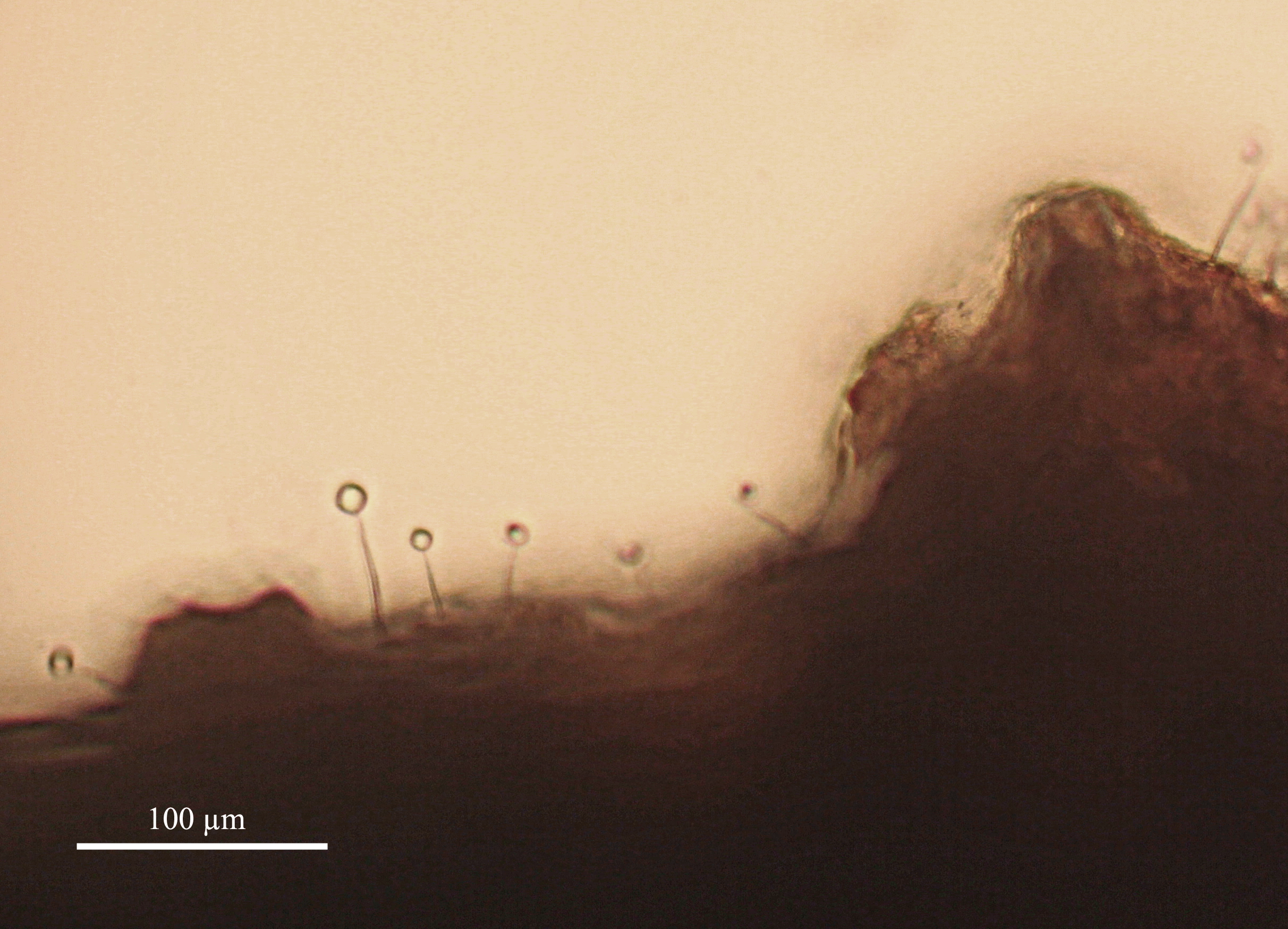

Supplement: Figure S1 — A cluster of sporocarps of the protosteloid amoeba Tychsporium acutostipes fruiting on a leaf. This image was taken at a total magnification of 100X. The scale bar is 100 µm. For high quality images of all species discussed in this paper, see Spiegel et al. (2007) online. [file peerj-02-296-s001.png]

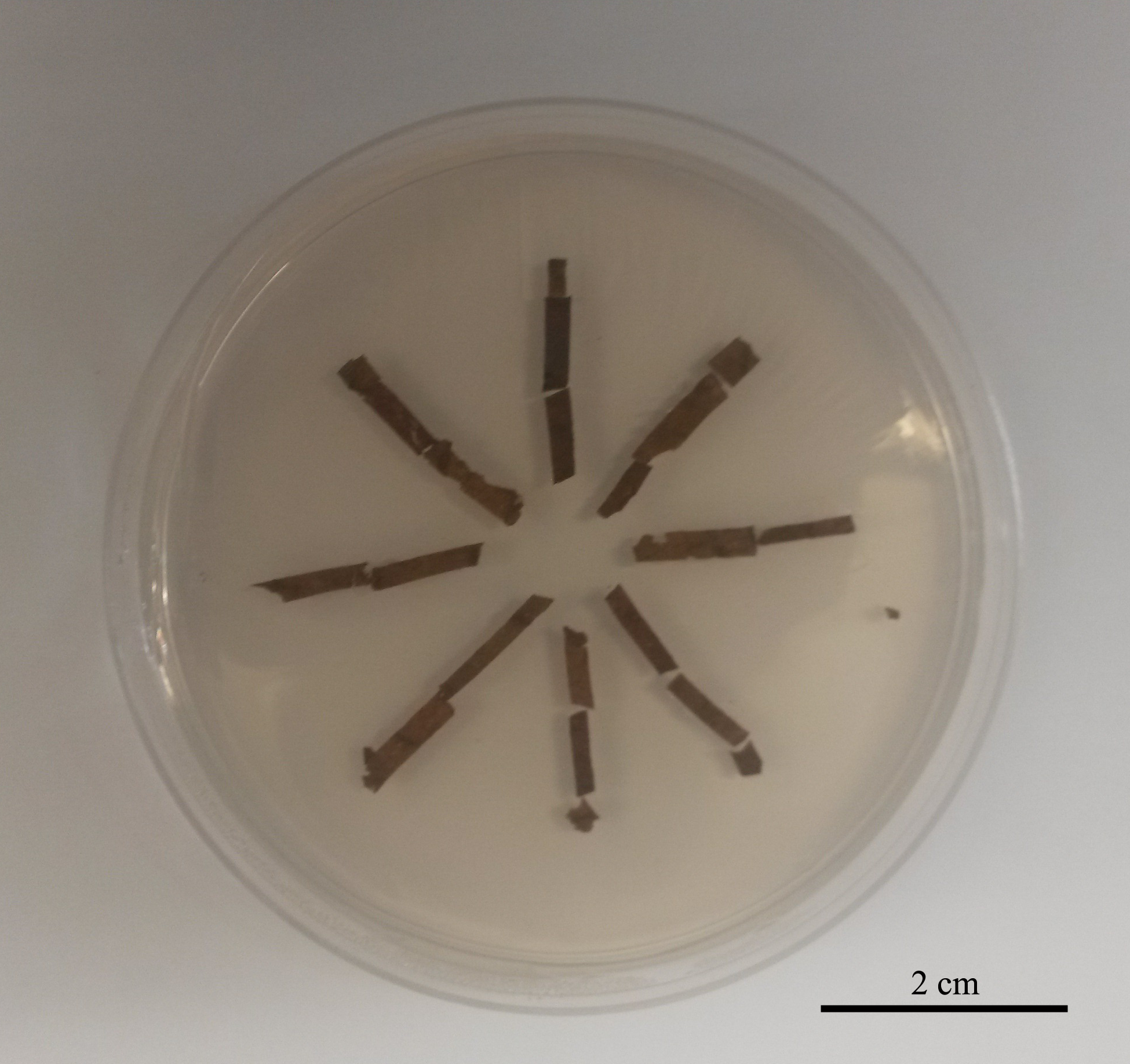

Supplement: Figure S2 — A primary isolation plate with 8 lines of substrate arranged in a circle. Each line of substrate is labeled and observations of protosteloid amoebae are labeled according to which line they occurred on. [file peerj-02-296-s003.png]
