# Supplementary material for: Ecological distribution of protosteloid amoebae in New Zealand
Source: PeerJ. 2014 Mar 11;2:e296. doi: 10.7717/peerj.296 (PMC3961141; doi:10.7717/peerj.296)
Supplement: Table S1 — Table of study sites. Habitat types are generalizations. No significant correlations between habitat type and abundance were found, either generally or by species. At some sites dead vegetation suitable as a substrate was very limited and at others it was highly abundant. Thus, the number of lines plated at each site varies from 4 to 443. [file peerj-02-296-s002.pdf]

Site	Latitude/Longitude	Elev. (m)	Habitat	Month/Year Collected	Lines Plated	Site Richness
Tairoa Head Albatross Colony (263)	45°46'30.1000"S, 170°43'41.4998"E	67	Grassland	3/2004	218	10
West of Dunback (264)	45°19'13.3000"S, 170°34'34.2001"E	130	Grassland	3/2004	306	13
West of Morrisons (265)	45°13'16.1000"S, 170°25'24.3001"E	561	Scrub	3/2004	192	11
Boundry Creek Rest Area (266)	44°21'13.5000"S, 169°10'07.7002"E	277	Mixed Dry Forest	3/2004	194	7
Blue Pools (267)	44°09'00.8640"S, 169°16'00.6100"E	277	Beech	3/2004	160	1
Haast Pass (268)	45°06'00.4380"S, 169°21'00.2830"E	716	Beech	3/2004	188	1
South of Haast (269)	44°03'21.1000"S, 168°42'35.3999"E	716	Rainforest	3/2004	320	7
Jacksons Head (270)	43°57'52.6000"S, 168°36'19.4000"E	1	Podocarp/Beech	3/2004	320	11
Road to Hokitika (271)	42°59'00.0790"S, 170°40'00.7961"E	30	Rainforest	3/2004	162	5
Port Elizabeth (272)	42°22'00.5920"S, 171°14'00.3862"E	0	Beach	3/2004	156	18
Punakaiki (273)	42°06'00.9560"S, 171°19'00.7741"E	0	Beach/Nileau	3/2004	336	9
Temple Basin Trail (274)	42°54'44.1000"S, 171°33'32.1001"E	876	Scrub	3/2004	160	7
The Chasin Trail (276)	42°55'09.3000"S, 171°33'30.4999"E	842	Beech	3/2004	162	1
U of Canterbury (277)	43°02'09.0000"S, 171°45'25.9999"E	561	Grassland	3/2004	168	6
Eastern Beech (278)	43°17'28.8000"S, 171°55'01.2000"E	493	Beech	3/2004	158	8
Sharplin Falls (279)	43°37'41.2000"S, 171°25'04.5998"E	463	Beech	3/2004	154	8
Peel Forest (280)	43°53'34.7000"S, 171°15'42.0001"E	289	Podocarp/Beech	3/2004	443	12
Te Anau (281)	45°26'38.0000"S, 167°41'03.0998"E	218	Beech	3/2004	229	3
Mirror Lake (282)	45°01'44.2000"S, 168°00'46.8000"E	350	Beech/Wetland	3/2004	239	2
Lake Gunn (283)	44°53'26.4000"S, 168°05'06.7999"E	485	Beech	3/2004	164	1
Red Tussock Conservation Area (284)	45°33'38.0000"S, 168°02'07.4000"E	480	Native Grassland	3/2004	162	6
Taputaputa Bay (302)	34°26'13.7400"S, 172°42'48.4200"E	5	Teatree	5/2005	40	10
Pine Block Road (303)	34°44'57.7800"S, 173°01'05.8800"E	70	Pine	5/2005	52	12
Ahipara Gum Lands (305)	35°11'40.6800"S, 173°08'06.5400"E	178	Teatree	5/2005	40	9
Herekino Forest Tracks (306)	35°12'35.5200"S, 173°11'27.2400"E	154	Teatree	5/2005	40	10
Mangamuka Forest (304)	35°11'24.2400"S, 173°27'18.7801"E	379	Broadleaf	5/2005	30	10
Puketi Forest (307)	35°16'32.6400"S, 173°41'09.9600"E	16	Podocarp	5/2005	40	13
Harrison Scenic Reserve (308)	35°18'37.2600"S, 174°06'24.7799"E	79	Forest (Coastal)	5/2005	40	9
Trounson Kauri Park (309)	35°43'13.5000"S, 173°39'00.1199"E	234	Podocarp	5/2005	40	1
Mill Bay (310)	36°59'30.7800"S, 174°36'11.2201"E	17	Rainforest	5/2005	44	5
Aratoro Scenic Reserve (359)	38°30'14.7420"S, 175°15'10.8000"E	129	Podocarp	12/2005	40	7
TongariroNP1 (360)	39°14'16.8540"S, 175°33'26.5680"E	1636	Scrub	12/2005	20	1
TongariroNP2 (361)	39°12'08.9820"S, 175°32'25.8720"E	1134	Beech	12/2005	40	6
DesertRoad (362)	39°18'59.4180"S, 175°43'49.7280"E	1015	Grassland	12/2005	40	2
TongariroNP3 (363)	39°10'10.6140"S, 175°31'26.5440"E	930	Flax/Scrub	12/2005	40	1
AraokiGorge (364)	38°40'16.8240"S, 174°41'40.1028"E	8	Tree Fern/Podocarp	12/2005	40	14
GorgePulloff (365)	38°53'45.9240"S, 174°35'56.4360"E	214	Tree Fern	12/2005	40	11
EgmontNp1 (366)	39°16'45.1560"S, 174°05'05.9280"E	1199	Scrub	12/2005	40	1
EgmontNP2 (367)	39°14'20.6880"S, 174°06'46.1160"E	941	Podocarp/Broadleaved	12/2005	40	2
EgmontNP3 (368)	39°18'28.4760"S, 174°05'50.2800"E	1159	scrub	12/2005	40	1

Site	Latitude/Longitude	Elev. (m)	Habitat	Month/Year Collected	Lines Plated	Site Richness
Wanganui1 (369)	39°49'08.7600"S, 174°50'22.2360"E	120	Mixed Broadleaf	12/2005	60	13
Wanganui2 (370)	39°45'54.2160"S, 175°10'15.1680"E	24	Beech	12/2005	40	10
Manawata (371)	40°20'22.5600"S, 175°49'05.3760"E	76	Broadleaf	12/2005	40	9
Waihini (372)	40°59'46.1760"S, 175°23'22.8120"E	166	Podocarp/Broadleaved	12/2005	40	3
Rimutaka (373)	41°20'56.3280"S, 174°56'15.9000"E	70	Podocarp/Broadleaved	12/2005	40	6
Titahi (374)	41°05'58.8840"S, 174°50'06.5760"E	0	Scrub (Coastal)	12/2005	40	9
QEPark (375)	40°58'19.5600"S, 174°57'36.5400"E	0	Scrub (Coastal)	12/2005	40	15
Otaki (376)	40°51'14.2920"S, 175°14'06.6480"E	128	Secondary Growth	12/2005	40	11
Mahia (377)	39°04'18.0480"S, 177°48'39.4920"E	34	Scrub	12/2005	40	10
Bush (378)	38°52'34.1040"S, 177°51'20.4480"E	543	Secondary Growth	12/2005	40	14
Okita (379)	38°39'53.5320"S, 178°10'49.4040"E	37	Mixed Broadleaf	12/2005	40	10
TeUruwera1 (380)	38°47'56.6880"S, 177°07'22.9440"E	607	Beech/Fern	12/2005	40	8
TeUruwera2 (381)	38°47'02.3280"S, 177°08'04.0200"E	609	Scrub	12/2005	40	14
TeUruwera3 (382)	38°43'43.8240"S, 177°05'11.0760"E	653	Beech/Podocarp	12/2005	40	11
TeUruwera4 (383)	38°39'51.3000"S, 177°02'13.3440"E	661	Beech	12/2005	40	6
HukaFalls (384)	38°38'57.3720"S, 176°05'20.6160"E	580	Broadleaf	12/2005	40	10
LakeTaupo (385)	38°44'41.7840"S, 176°04'07.5000"E	367	Grassland	12/2005	40	7
HinaKapu (386)	38°02'14.6400"S, 176°33'00.0000"E	350	Podocarp	12/2005	40	9
BayPlenty (387)	37°52'15.2400"S, 176°42'32.0400"E	2	Dunes	12/2005	40	4
Hiwy25 (388)	37°18'16.9920"S, 175°53'29.7600"E	65	broadleaf	12/2005	40	9
TwinKauri (389)	36°58'44.6520"S, 175°50'30.9120"E	117	Tree Fern/Kauri	12/2005	40	10
Maungataururu (390)	36°44'54.7440"S, 175°32'15.2520"E	370	Tree Fern/Nikau	12/2005	40	12
SquareKauri (391)	36°59'23.0640"S, 175°34'19.3080"E	306	Kauri/Broadleaved	12/2005	40	9
Hihi (392)	37°06'43.5600"S, 175°38'02.2920"E	59	Nikau/Broadleaved	12/2005	40	11
AUK06-1 (422)	50°50'20.6412"S, 165°55'15.2400"E	9	Forest (Coastal)	3/2006	4	2
AUK06-2 (423)	50°50'20.6412"S, 165°55'15.2400"E	9	Forest (Coastal)	3/2006	4	2
AUK06-4 (425)	50°51'11.0412"S, 165°55'12.9000"E	324	Forest (Coastal)	3/2006	4	1
AUK06-9 (430)	50°48'58.6188"S, 166°12'02.5200"E	20	Forest (Coastal)	3/2006	4	2
AUK06-16 (437)	50°32'43.8612"S, 166°12'45.7812"E	11	Forest (Coastal)	3/2006	4	1
AUK06-17 (438)	50°29'34.3788"S, 166°16'51.9600"E	35	Scrub (Coastal)	3/2006	4	3
AUK06-19 (440)	50°31'51.4812"S, 166°18'05.1588"E	6	Scrub (Coastal)	3/2006	4	1
AUK06-20 (441)	50°31'51.4812"S, 166°18'05.1588"E	6	Scrub (Coastal)	3/2006	4	1
Charming Creek (1188)	41°44'24.0000"S, 171°35'42.0000"E	3	Forest (Native)	5/2006	24	1
Truman Track (1187)	42°00'38.8800"S, 171°20'09.6000"E	0	Scrub (Coastal)	5/2006	20	2
Knight's Bush (1281)	45°54'44.1000"S, 169°29'42.5004"E	152	Beech/Broadleaved	5/2007	20	8
Route 6 Nelson (1282)	41°09'47.4984"S, 173°32'55.3992"E	84	Scrub	5/2007	20	1
Kowhai Point (1284)	41°42'44.2008"S, 173°06'46.2996"E	420	Scrub	5/2007	20	5
Lewis Pass (1286)	42°22'26.4000"S, 172°23'46.7988"E	914	Beech	5/2007	16	1
Route 63 (1287)	42°01'52.1004"S, 172°14'35.8008"E	479	Beech	5/2007	16	3
Kahurangi (1288)	41°41'07.5984"S, 172°26'37.1004"E	259	Beech/Broadleaved	5/2007	16	4
Pigeon Saddle (1289)	40°49'57.2988"S, 172°58'08.5008"E	244	Tree Fern/Broadleaved	5/2007	32	6
