# Supplementary material for: Ecological distribution of protosteloid amoebae in New Zealand
Source: PeerJ. 2014 Mar 11;2:e296. doi: 10.7717/peerj.296 (PMC3961141; doi:10.7717/peerj.296)
Supplement: Table S2 — Kruskal-Wallis test statistics and P-values for the influence of environmental factors on protosteloid abundance and richness. Model = Response × Factor. Abundance refers to scaled abundance per line of substrate. Richness refers to scaled richness per line of substrate. Test statistics are corrected for ties. All models showed significant effects of environmental gradients on scaled abundance and richness. [file peerj-02-296-s004.docx]

| **Model** | **Test Statistic (H)** | **P-value** |
| --- | --- | --- |
| Abundance x Distance from equator | 341.38 | <0.0005 |
| Abundance x Elevation | 264.68 | <0.0005 |
| Abundance x Precipitation | 275.23 | <0.0005 |
| Richness x Distance from equator | 298.86 | <0.0005 |
| Richness x Elevation | 248.29 | <0.0005 |
| Richness x Precipitation | 259.39 | <0.0005 |
